# TNFα activation and TGFβ blockage act synergistically for smooth muscle cell calcification in patients with venous thrombosis via TGFβ/ERK pathway

**DOI:** 10.1111/jcmm.17472

**Published:** 2022-07-08

**Authors:** Penghui Wang, Yiqing Pan, Chenghao Yang, Linjie Zhang, Zhen Zhao, Kaichuang Ye, Lei Li, Shoubing Xia, Xinwu Lu, Huihua Shi, Weimin Li, Minyi Yin

**Affiliations:** ^1^ Department of Vascular Surgery, Shanghai Ninth People's Hospital Shanghai JiaoTong University School of Medicine Shanghai China; ^2^ Department of Histoembryology, Genetics and Developmental Biology, Shanghai Key Laboratory of Reproductive Medicine Shanghai Jiao Tong University School of Medicine Shanghai China; ^3^ Vascular Center of Shanghai JiaoTong University Shanghai China

**Keywords:** calcification, phenotypic plasticity, TGF‐beta, TNF‐alpha, venous thrombosis

## Abstract

Venous calcification has been observed in post‐thrombotic syndrome (PTS) patients; yet, the cell types and possible mechanisms regulating this process are still unclear. We evaluated the calcium deposition within the venous wall, the cell type involved in the calcified remodelling of the venous wall after thrombosis and explored possible mechanisms in vitro. Calcium deposition was found in human specimens of superficial thrombotic veins and was co‐localized with VSMCs markers αSMA and TAGLN (also known as SM22α). Besides, the expression of osteogenesis‐related genes was dramatically changed in superficial thrombotic veins. Moreover, the inhibition of the TGFβ signalling pathway after TNFα treatment effectively induced the expression of osteogenic phenotype markers, the calcium salt deposits and the obvious phosphorylation of ERK1/2 and JNK2 in the VSMCs calcification model. Supplementing TGFβ2 or blocking the activation of the ERK/MAPK signalling pathway prevented the transformation of VSMCs into osteoblast‐like cells in vitro. Taken together, VSMCs have an important role in venous calcification after thrombosis. Supplementing TGFβ2 or inhibiting the ERK/MAPK signalling pathway can reduce the appearance of VSMCs osteogenic phenotype. Our findings may present a novel therapeutic approach to prevent of vascular calcification after venous thrombosis.

## 1 INTRODUCTION

Deep vein thrombosis (DVT) is a common disease with potentially devastating and long‐term sequelae. It usually occurs when a blood clot forms in a deep vein. The main sequelae of DVT are pulmonary embolism (PE) and post‐thrombotic syndrome (PTS).^1^ Even with early and timely regular anticoagulation treatment, up to one‐half of patients with DVT develop PTS, whose typical clinical manifestations include pain, swelling, heaviness, fatigue, itching and cramping in the affected limb^2^; this, in turn, leads to increased healthcare costs and reduced quality of life.^3^, ^4^ The mechanisms of PTS mainly consist of persistent venous outflow restriction or obstruction, vein wall remodelling^5^ and valvular reflux,^6^ eventually resulting in venous hypertension.^7^ Recent studies have shown that even if the thrombus burden was completely removed (after 8 days of thrombosis), it might not prevent the development of PTS, which suggested that vein wall remodelling after vein wall damage has a vital role in the presence and development of PTS.^8^

Calcified remodelling of the venous wall is a type of vein wall remodelling leading to chronic venous hypertension. Several studies reported its influence on chronic venous insufficiency (CVI).^9^, ^10^ Even though calcified iliac venous and external iliac venous thrombosis have been described from the lower extremity venograms in 1975,^11^, ^12^ the underlying mechanism of the process remains unclear. So far, a few cases reports suggested a link between venous calcification and recurrence of deep venous thrombosis and pulmonary embolism.^13^, ^14^ Besides, calcification related to in‐stent restenosis in a venous stent has been also reported.^15^ Therefore, further research in investigating the underlying mechanism of venous calcification after DVT has critical clinical significance for preventing and treating PTS.

Vascular smooth muscle cells (VSMCs) seem to have an important role in the initiation and progression of vascular calcification. VSMCs can adapt to several phenotypes after exposure to multiple growth factors and inflammatory mediators, including adipogenic, macrophagic and calcific (osteogenic, chondrocytic and osteoclastic) phenotypes. VSMCs dedifferentiation from the contractile phenotype to the osteoblast‐like cells, also known as a phenotypic switch, is thought to occur prior to vascular diseases.^16^ Diverse conditions interfere with this process, including inflammation, loss of calcification inhibitors, oxidative stress, mitochondrial dysfunction and disturbed calcium and phosphate homeostasis, which drive VSMCs to acquire osteogenic phenotype. During dedifferentiation from contractile phenotype into osteoblast‐like cells, VSMCs express high levels of osteogenic markers (e.g. Runx2, osterix, osteocalcin, osteopontin and alkaline phosphatase (ALP)) which facilitate the initiation and development of arterial calcification.^17^ Though the function of VSMCs phenotypic switch in arterial diseases is relatively clear, its role towards venous disease, especially in venous calcification of PTS, is not fully elucidated.

In this study, we examined the association between the VSMCs phenotypic switch and venous calcification of PTS and the mechanism involved in this process.

## 2 MATERIALS AND METHODS

### 2.1 Human vein wall tissue collection

The Institutional Ethics Committee of Shanghai Ninth People's Hospital, School of Medicine, Shanghai Jiao Tong University approved this study (ethics number: 2016‐Num. 8). The vein wall tissue from patients with superficial thrombophlebitis was selected (*n* = 11). Inclusion criteria for patients with superficial thrombophlebitis were 1. patients with varicose veins combined with superficial phlebitis of the lower extremities, typically manifesting as localized swelling, pain (aggravated after walking) and palpable cord overlying the track of a superficial vein; 2. preliminary occurrence of superficial thrombophlebitis, which lasted more than 2 weeks; 3. ultrasound showing slight hyperechoic structure with intraluminal echogenicity; thickness of the localized venous wall, and recanalization of irregular, small branch‐like blood flow within the thrombus. Exclusion criteria were 1. combined with systemic connective tissue diseases, such as Marfan syndrome; 2. superficial thrombophlebitis caused by injury; 3. thrombus in the whole superficial varicose veins; lack of non‐thrombotic superficial veins as an internal comparison; 4. patients with acute (less than 2 weeks) or recurrent superficial thrombophlebitis. The patient demographics are listed in Table S1.

### 2.2 Computed tomography venography (CTV)

All PTS patients underwent Computed tomography venography (CTV) using a 64‐detector row CT scanner (GE Healthcare, Boston, MA, USA). The protocol of CTV examination was previously described.^18^

### 2.3 Microarray‐based gene expression analysis

Total RNA of human vein wall tissues was isolated using the TRIzol® reagent (Invitrogen). Each sample was assessed using Agilent Bioanalyzer 2100 (Agilent Technologies) for RNA integrity. Only RNAs with OD260/OD280 > 1.8 and an RNA integrity number (RIN) > 8 were used for microarray experiments.

### 2.4 Animals

Male Sprague–Dawley rats (100~150 g for separation of VSMCs; 200~250 g for construction of rat vein thrombosis model) that were used for all studies were obtained from Shanghai JiaoTong University, School of Medicine. All the animals were housed in an environment with a temperature of 22 ± 1°C, relative humidity of 50 ± 1%, and a light/dark cycle of 12/12 h. All animal studies (including the rats euthanasia procedure) were done in compliance with the regulations and guidelines of Shanghai JiaoTong University, School of Medicine institutional animal care and conducted according to the AAALAC and the IACUC guidelines.

### 2.5 Antibodies and reagents

U0126, JNK inhibitor II (Calbiochem Corp, USA) and SB505124 (Selleck, USA) were dissolved in DMSO (final concentration lower than 0.1%). The cytokines of TNFα and TGFβ2 were from Peprotech and Selleck, respectively. Dulbecco's modified Eagle's medium (DMEM), penicillin and streptomycin were purchased from Invitrogen Life Technologies. Antibodies of phospho‐ERK1/2 (Thr202/Tyr204), phospho‐JNK (Thr183/Tyr185), phospho‐P38 (Thr180/Tyr182), phospho‐Smad2 (Ser465/467)/Smad3 (Ser423/425), total P38, SMAD2/3 and Runx2 were from Cell Signalling Technology. COL1A1, αSMA and TAGLN were bought from Abcam. Total‐ERK2 was bought from Santa Cruz Biotechnology, and α‐tubulin‐HRP antibody was obtained from Proteintech Group. All other reagents were purchased from Sigma‐Aldrich unless indicated otherwise. Detailed information of the antibodies is listed in Table S2.

### 2.6 Separation and culture of rat VSMCs

Primary rat VSMCs were isolated and cultured using previously described methods.^19^ Primary VSMCs were grown in a humidified incubator (Thermo Fisher Scientific) at 37 °C in a 5% CO_2_ atmosphere, and VSMCs between the 3rd and 8th passages were used for all experiments.

### 2.7 Osteoblastic differentiation of rat VSMCs

Before cells were plated, 12 (or 24) well plates were incubated with 1% gelatin (diluted with DPBS at the ratio of 1:1) and then transferred into an incubator at 37 °C for at least 15 min. After removing the medium, VSMCs were seeded at a density of 5 × 10^4^ (or 2.5 × 10^4^) per well in DMEM supplemented with 5% FBS. Twenty‐four hours later, cells were treated with osteogenic medium (OM), containing DMEM supplemented with CaCl_2_ (4 mM), β‐glycerophosphate (5 mM), L‐ascorbic acid (50 μg/mL), insulin (1 μM), dexamethasone (0.1 μM) and 5% FBS. In order to induce dedifferentiation of VSMCs, TNFα (20 ng/mL) was added to each well. A day later, a cell medium was replaced with a medium containing OM with or without TNFα (20 ng/mL), SB505124(1 μM) or SB505124(2.5 μM), respectively. The medium was changed each day.

### 2.8 Surgical model of rat deep vein thrombosis

Thrombosis was induced by inferior vena cava (IVC) ligation as previous described.^20^ Briefly, SD rats were anaesthetised by inhaling isoflurane, then the IVC were exposed. Any lateral or lumbar branches draining into the IVC between the left renal vein and the iliac bifurcation are interrupted, and the IVC is completely ligated. At days 14, pluronic gel (Sigma‐Aldrich) with or without TNFα (25 μg/kg) and/or SB505124(5 mg/kg) was applied to the adventitial surface of rat IVC. Veins with thrombi were harvested after treated for 24 or 48 h for detection the expression of osteogenic‐related genes.

### 2.9 Alizarin Red S staining

After 8 days of culture, VSMCs were fixed in 4% paraformaldehyde for 30 min at 4°C, rinsed in dH_2_O and incubated with alizarin red solution (Solarbio, Beijing, China) for 5 min to visualize deposition of calcium. Images were taken and analysed. Human vascular tissues were fixed in 4% formalin, paraffin‐embedded and 5 μm‐thick transverse sections out, deparaffinized and stained using Alizarin red solution to visualize calcium deposition.

### 2.10 Western blot

Cells were lysed with lysis buffer (10 mM Tris/100 mM NaCl/5 mM EDTA/10% Glycerol/1% triton x‐100/1% NP‐40/0.1% SDS/ Protein Inhibitor Cocktail 1:100) and then detected using a Bradford method to quantify its protein concentration. Consequently, 30 or 40 μg of lysate protein was run on 10% SDS‐PAGE gel and transferred to the PVDF membrane. The membranes were blocked in 5% non‐fat milk dissolved in 1 × TBS buffer at room temperature for 1 h and then incubated with the primary antibody at 4°C overnight. After being washed three times in TBST, the blots were incubated with horseradish peroxidase (HRP)‐coupled secondary antibodies at a dilution of 1:10000 for 1 h at room temperature. Western blot analysis was conducted by using ECL reagent (Pierce) and detected using image analysis software (Bio‐Rad Laboratories). The membranes were re‐probed with internal reference antibodies, such as ERK2, P38, Smad2/3 or Tubulin, after probed with antibodies against phospho‐ERK1/2, phospho‐JNK, phospho‐P38, phospho‐Smad2/Smad3 or αSMA, TAGLN and COL1A1. The expression of the protein was normalized to tubulin and expressed as fold change of control.

### 2.11 Quantitative PCR analysis

Total RNA from the indicated tissues or cells was homogenized in TRIzol® reagent (Invitrogen). RNA was reversely transcribed, and real time quantitative PCR was conducted using a SYBR Green master mix and a 40‐cycle thermocycling protocol. The data are presented as relative fold changes in mRNA normalized to GAPDH. The primer sequences are given in Table S3.

### 2.12 Immunofluorescence staining

Cultured VSMCs were fixed in 4% paraformaldehyde at 4°C for 40 min. For human samples, 8~10 μm frozen sections were prepared and fixed with 4% paraformaldehyde. Then, sections and cultured VSMCs were permeabilized and blocked by 0.1% Triton X‐100 in 5% bovine serum albumin at room temperature for half an hour, followed by a subsequent overnight incubation at 4°C with primary antibodies (Runx2, αSMA, TAGLN with 1:100 dilution). After washing, the sections or VSMCs were incubated with secondary antibodies which were conjugated with either Alexa fluor® 488 or Alexa fluor® 555 (Invitrogen) at 1:500 dilution for 1 h at room temperature. DAPI was used to stain nuclei in PBS for 10 min. Finally, the fluorescence‐stained sections were viewed under a fluorescence microscope (Nikon, Japan).

### 2.13 Statistical analysis

All quantitative results are means ± SEM unless specified (*n* ≥ 3). GraphPad Prism software (Version 8.0.1) was used for statistical analysis. Differences between groups were tested by Student's *t* test for unpaired means. Fisher's exact test was used to analyse the differential expression of osteogenic markers in human specimens. A *p* value <0.05 was considered statistically significant.

## 3 RESULTS

### 3.1 Venous calcification in human thrombosis vein walls

**FIGURE 1:**
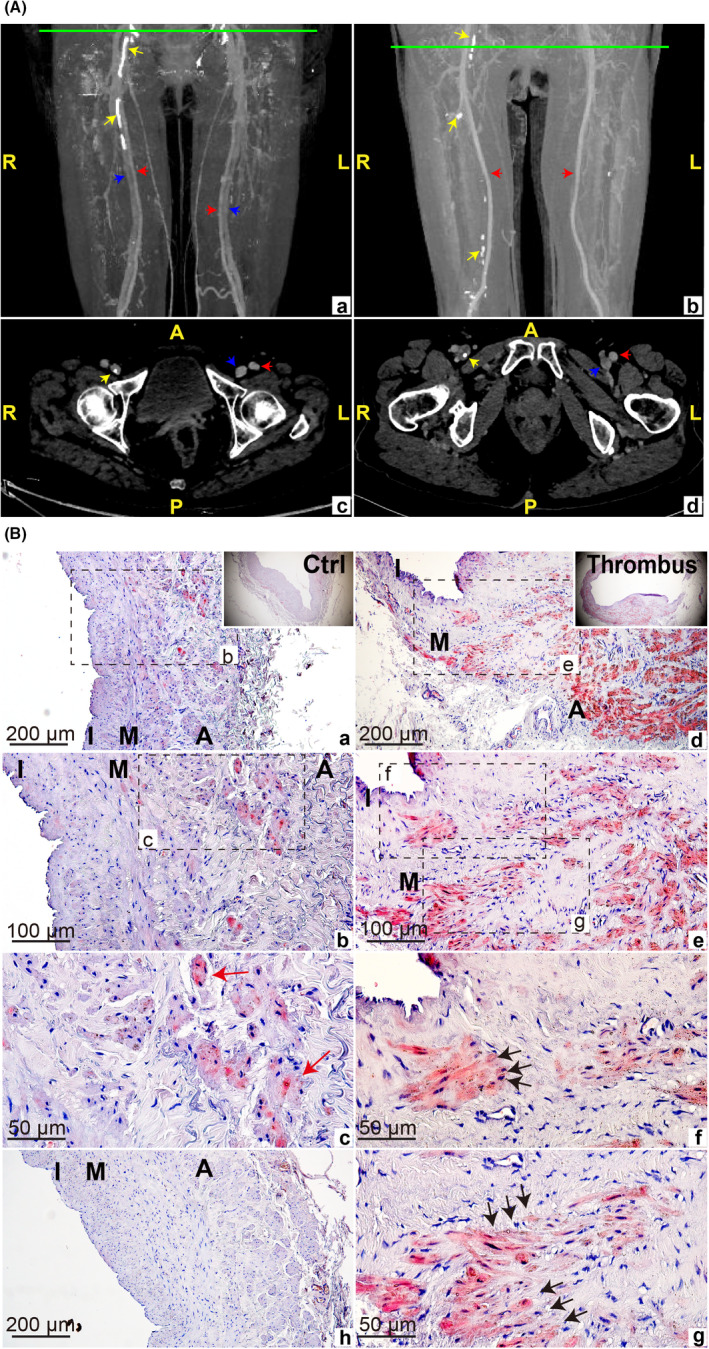
Venous calcification in human thrombosis vein walls. A: Representative CTV images of venous calcification of PTS patients. (A, B) were coronal images. (C, D) were axial images. Red arrows marked arterials and blue arrows signed the veins. As visualization of the deep vein adjacent to the arterial of image b was obscure, no signs were marked the deep vein. The yellow arrows indicated venous calcification. The green lines marked the section site of axial plane in coronal images. A: anterior, P: posterior, L: left, R: right. B: a–c and d–g showed Alizarin Red S staining of non‐thrombotic (as internal comparison) and thrombotic venous wall in different magnification, respectively. h represented Alizarin Red S staining of robust vein wall tissue, as a negative control. I: intimal, M: medial, A: adventitial

During the treatment, we initially observed that obvious calcification spots could be detected in the venous wall from CTV of PTS patients (Figure 1A), suggesting that the calcified remodelling of the venous wall may be involved in the occurrence and development of PTS. We collected human thrombotic and non‐thrombotic superficial vein wall specimens after venous thrombosis 0.5 to 2 months, during which patients exhibited a high incidence of vessel wall remodelling. In order to clarify whether venous thrombosis could promote the appearance of venous wall calcification, we measured calcium deposition in the venous wall by using Alizarin Red S staining. Compared with the non‐thrombotic vein wall, calcium deposition was significantly increased in the thrombotic vein wall (Figure 1B). In addition, unlike the arterial calcification that mainly manifests as intima or medial calcification, the site of calcium deposition within the venous wall after thrombosis was mainly gathered at the media and adventitia, and the spotty calcification in the media presented spindle shape, similar to the morphology of VSMCs (black arrows). We assumed that calcium deposition was caused by VSMCs, which subsequently gave rise to the decreased compliance of the vein wall and further exacerbated damage to the wall vein after thrombosis.

### 3.2 VSMCs are involved in venous calcification in patients with venous thrombosis

**FIGURE 2:**
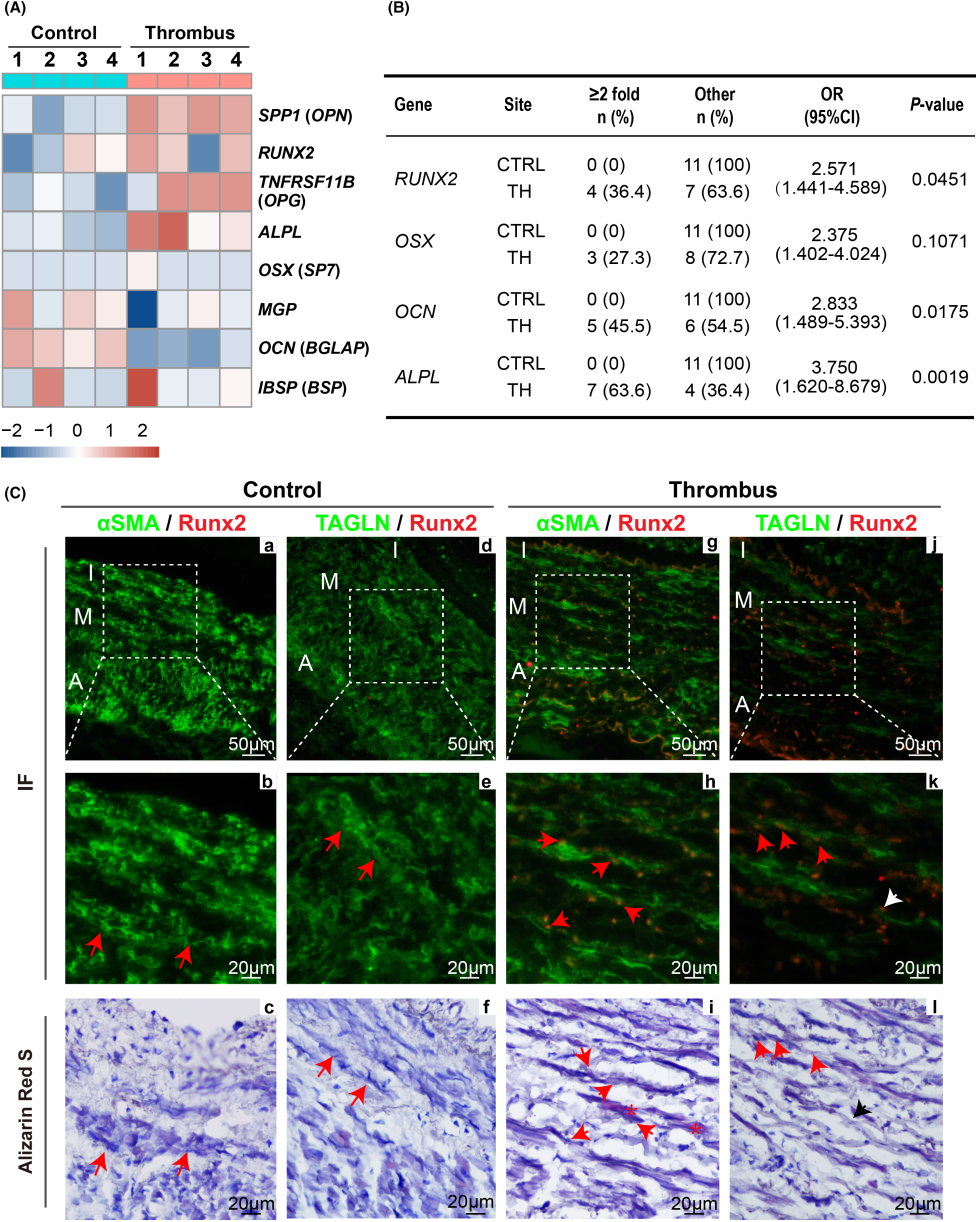
Expressions and distributions of osteogenic markers (RUNX2, ALPL and OCN) in patients with venous thrombosis. (A) Heatmap of osteogenesis‐related genes in human control and thrombotic vein wall. (B) Thrombotic and non‐thrombotic venous tissues were harvested for RNA, qPCR was carried out using primers specific to *RUNX2*, *OSX*, *ALPL* and *OCN* (*n* = 11). (C) Samples were immunostained with the anti‐RUNX2, anti‐αSMA or anti‐TAGLN antibodies, RUNX2 (red fluorescence) was found to be co‐localized with αSMA or TAGLN (green fluorescence) in thrombotic vein wall. *p < 0.05, **p < 0.01; ns, non‐significant

To explore the molecular mechanism related to calcification of the post‐thrombotic venous wall, we applied microarray‐based gene expression analysis to compare the differentially expressed genes in 4 pairs of human thrombotic and non‐thrombotic superficial veins specimens and specifically analysed the expression of genes that are associated with the osteogenesis process. The heatmap showed that a part of genes regulating the osteogenesis process was changed (Figure 2A). To verify the heatmap results, we investigated changes in osteogenesis‐related genes of the vein wall in the mRNA level and found osteogenic transcription factors (*RUN2* and *OSX*) and osteogenic‐specific proteins (*ALPL* and *OCN*) increased in a part of thrombotic superficial vein wall specimens (Figure 2B). The above results implied that calcification might occur in the thrombotic vein wall at the early stage of venous thrombosis.

Moreover, previous studies have shown that various types of cells participate in vascular calcification, including VSMCs, endothelial cells, fibroblasts, pericytes, mesenchymal stem cells and progenitor cells.^21^ Thus, we next investigated which cell type may be involved in the calcium deposition in the vein wall after thrombosis using immunofluorescent staining combined with Alizarin Red S staining of human specimens (Figure 2C). Compared with non‐thrombotic veins, calcium deposits were observed in the thrombotic vein wall, and Runx2 signals were found at the calcium distribution site. Besides, intracellular localization of Runx2 was partially overlapped with the distribution of αSMA‐positive spindle‐shaped VSMCs. Staining with the VSMCs differentiation marker TAGLN was further performed, revealing that αSMA and TAGLN double‐positive VSMCs located at calcium deposits area also expressed Runx2 (red arrow in Figure 2C–H, K), which suggested that VSMCs were involved in the calcification process of the venous wall after thrombosis. Furthermore, cells expressing Runx2 with light TAGLN signals were seen close to VSMCs (white arrow in Figure 2C–K). Based on this, we speculated that the phenotypic switch of VSMCs influences the appearance of calcification. The above results suggested that VSMCs in the media of the venous wall may have a vital role in the calcified remodelling process of the venous wall after thrombosis via a phenotypic switch.

### 3.3 TNFα activation together with TGFβ downregulation induces VSMCs phenotypic switching in vitro

**FIGURE 3:**
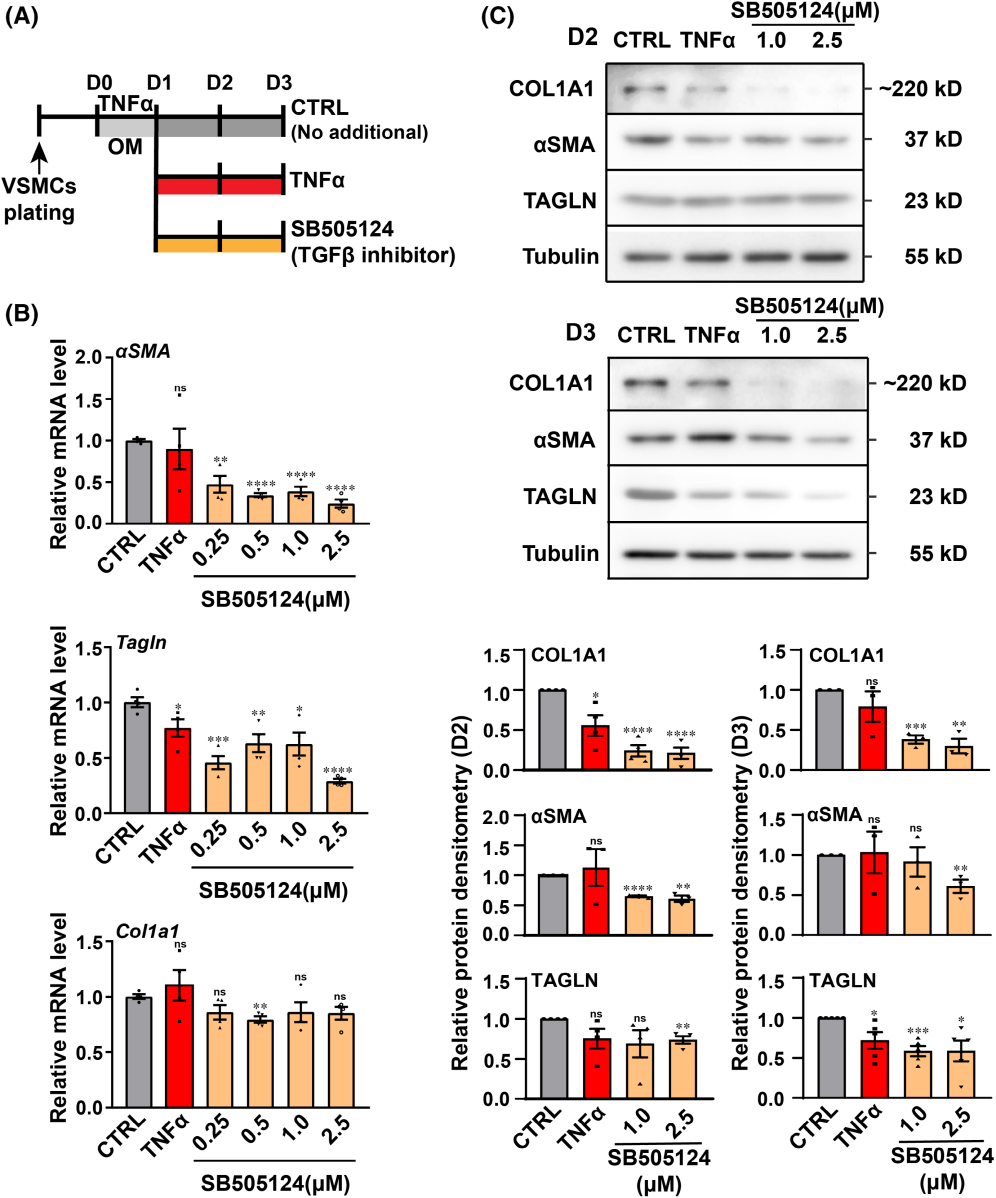
Expressions of phenotypic markers induced by TNFα activation and TGFβ downregulation in rat VSMCs. (A) Schematic diagram showing the strategy of inducing rat primary VSMCs calcification in vitro. (B) Transcription level of VSMCs contractile markers (*αSMA* and *Tagln*) and synthetic marker (*Col1a1*) after 2 days of culture in OM. (C) VSMCs were cultured in OM for 2 or 3 days, cell lysates were subjected to Western blot analyses for αSMA, TAGLN and COL1A1. The protein levels were quantified by ImageJ. *p < 0.05, **p < 0.01, ***p < 0.001, ****p < 0.0001; ns, non‐significant; *n* ≥ 3

To explore further the role of VSMCs in the calcification of venous vessel walls, we tried to construct a VSMC calcification model in vitro. In our previous studies, the expression of the TNFα signalling pathway in the superficial vein wall after thrombosis apparently increased; however, the TGFβ signalling pathway was suppressed. These two signalling pathways may be closely related to the VSMC phenotypic switch from the contractile to the synthetic phenotype. Besides, VSMC can alter their phenotype in response to different local cues and display features of chondrocytes, osteoblasts, adipocytes and macrophage foam cells.^22^ Based on this, we speculated that these two signalling pathways might also regulate the trans‐differentiation of VSMCs to osteoblast‐like cells. We firstly treated VSMCs with osteogenic medium (OM) containing TNFα and SB505124, respectively, and found that the mRNA level of osteoblast cell marker (*Runx2*) did not change. Previous studies have reported that robust contractile VSMCs are not sensitive to calcifying conditions; however, the situation could be different when cells undergo dedifferentiation.^23^ Thus, in this study, we treated VSMCs with TNFα in OM for 1 day to promote VSMCs loss of contractile phenotypic markers and then utilized SB505124 (a selective inhibitor of TGFβ type I receptors) with OM to induce VSMCs to transdifferentiate into an osteogenic phenotype (Figure 3A). In the SB group, we observed that the expression of typical contractile SMC markers (*αSMA* and *Tagln*) was significantly downregulated at the mRNA and protein levels and the hallmark of synthetic VSMCs (*Col1a1*) decreased at the protein level (Figure 3B–C). The results indicated that VSMCs have already undergone dedifferentiation under this culture condition.

### 3.4 TNFα activation and TGFβ blockage act synergistically to induce VSMCs calcification in vitro

**FIGURE 4:**
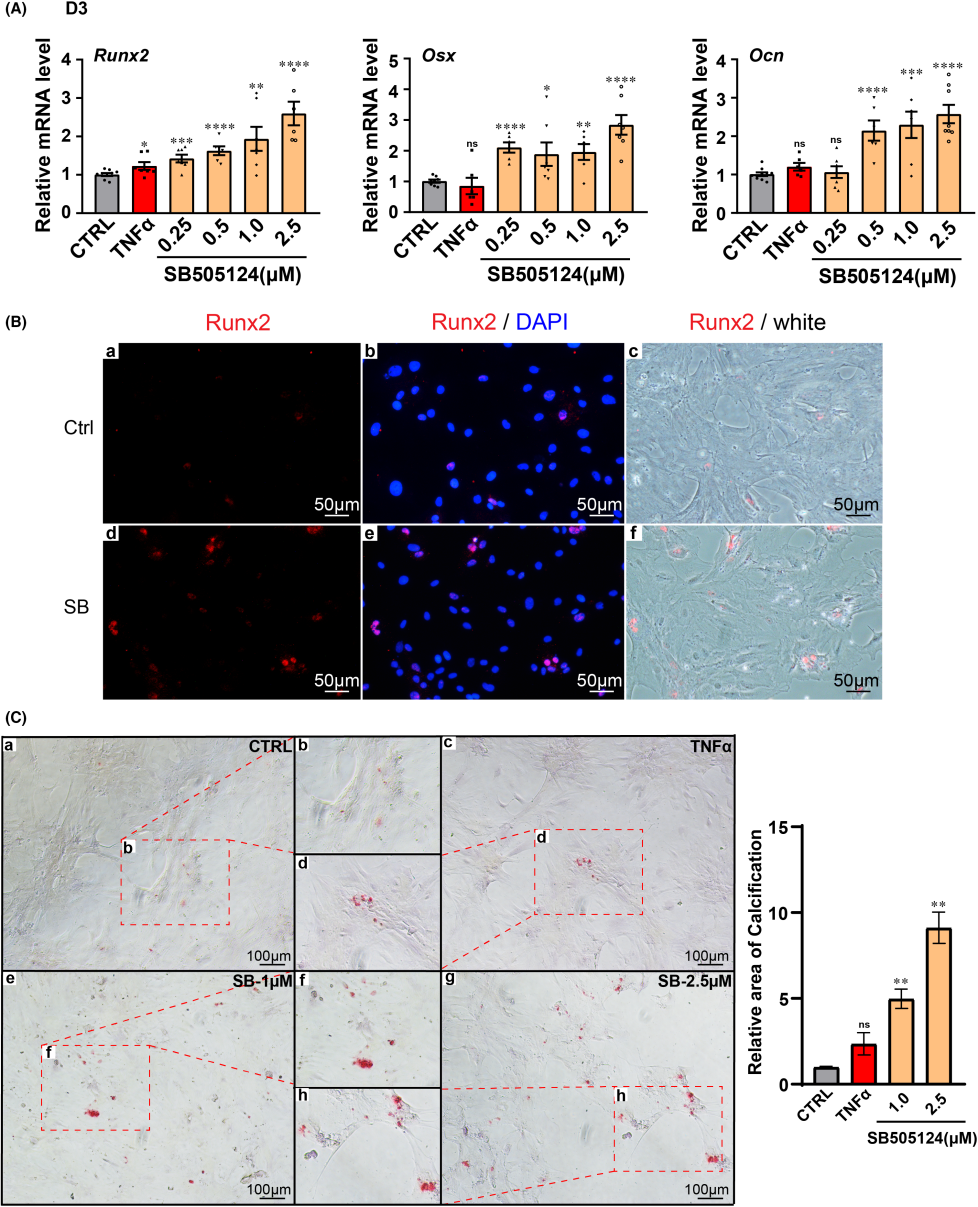
Expressions and distributions of osteogenic markers by TNFα activation and TGFβ downregulation in rat VSMCs. (A) qPCR was performed for VSMCs osteogenic markers (*Runx2*, *Osx* and *Ocn*) after 3 days culture in OM. (B) Cells were immunostained with an anti‐RUNX2 antibody and DAPI, RUNX2 (red fluorescence) was visualized in part of VSMCs of SB group. (C) Alizarin Red S staining showed calcium deposition in SB group. *p < 0.05, **p < 0.01, ***p < 0.001, ****p < 0.0001; ns, non‐significant; *n* ≥ 3

In an attempt to verify whether VSMCs dedifferentiated into an osteogenic phenotype, we inspected the changes in osteogenic specific transcription factors (*Runx2* and *Osx*) and osteogenic‐specific protein (*Ocn*) at the mRNA level. The results revealed strong upregulation of *Runx2* in the SB group, as well as *Osx* and *Ocn* (Figure 4A). Consistent with the mRNA results, the immunofluorescence showed the expression of Runx2 went up in the SB group (Figure 4B). The present results strongly suggested that VSMCs dedifferentiate and transform into osteoblast‐like cells. So far, we have verified that by activating the TNFα signalling pathway and inhibiting the TGFβ signalling pathway, VSMCs could be effectively induced by dedifferentiation into an osteogenic phenotype. Furthermore, Alizarin Red S staining showed that the calcium deposit was affected in the SB group (Figure 4C). Taken together, these results demonstrated that stimulating VSMCs with TNFα can reduce contractile phenotypic markers expression, subsequently inhibiting the TGFβ signalling pathway to promote VSMCs to gain osteogenic phenotype markers and to accelerate the rate of calcium deposition. It was suggested that the combined effect of TGFβ and TNFα signalling pathways might be an important regulatory factor for the transformation of VSMCs to osteoblast‐like cells in the early stage of PTS.

### 3.5 TNFα activation and TGFβ downregulation activate non‐canonical TGFβ/MAPK signalling molecules in vitro

**FIGURE 5:**
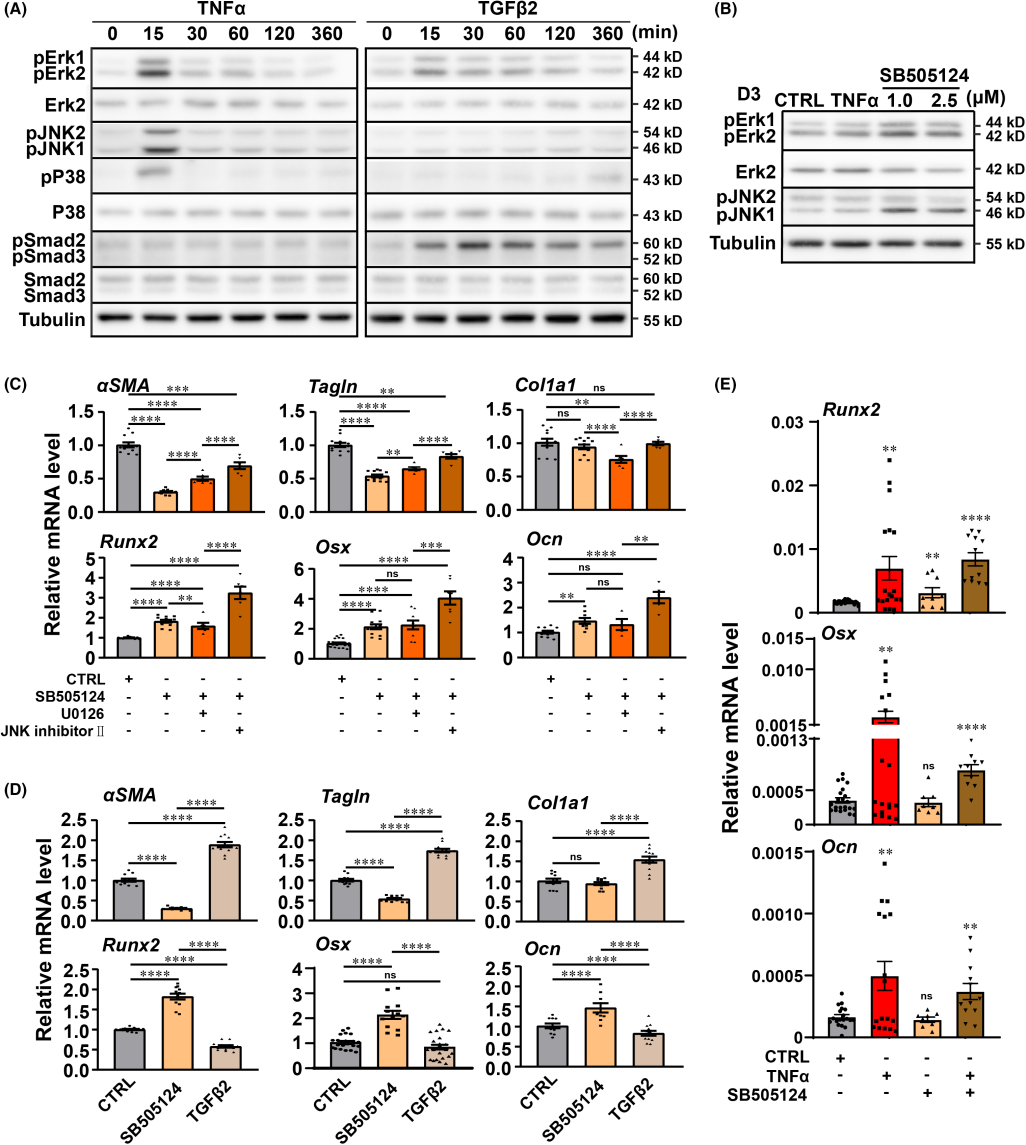
Involvement of ERK/MAPK molecule and the effects of TGFβ2 compensation or ERK/MAPK blockage on VSMCs calcification. (A) VSMCs were either not stimulated or stimulated with TNFα or TGFβ2 for indicated time. Cell lysates were subjected to Western blot analyses for phospho‐ERK1/2 (ERK2), phospho‐JNK1/2, phospho‐P38 (P38), phospho‐Smad2/3 (Smad2/3) and tubulin. (B) VSMCs were incubated with TNFα or SB505124 as indicated time. Cell lysates were subjected to Western blot analyses for phospho‐ERK1/2 (ERK2), phospho‐JNK1/2 and tubulin. (C) Cells were treated with SB505124, U0126 or JNK inhibitor II and phenotypic or osteogenic markers of VSMCs were performed by real‐time analyses. (D) Cells were treated with SB505124 or TGFβ2 and phenotypic or osteogenic markers of VSMCs were performed by qPCR analyses. (E) Detection of the osteogenic markers in rat thrombosis vein walls by real‐time PCR analyses. *p < 0.05, **p < 0.01, ***p < 0.001, ****p < 0.0001; ns, non‐significant; *n* ≥ 3

In vitro experimental results revealed that TNFα and TGFβ signalling pathways are closely related to VSMCs calcification; yet, the molecular mechanism is still unclear. Inflammation is closely associated with vascular calcification. The inflammatory factor TNFα is capable of enhancing in vitro vascular calcification by activation of the osteogenic process of VSMCs.^24^ In human specimens, we observed a substantial elevation of TNFα signalling in the vessel wall. In the in vitro model, we confirmed that VSMCs treated with TNFα went through a dramatically phenotypic switch. Previous studies have also shown that TNFα can effectively activate the MAPK signalling pathway by phosphorylating ERK, JNK, P38 and participating in the regulation of cell survival, proliferation and differentiation.^25^ Therefore, we chose to do the time‐course study by stimulating VSMCs with TNFα in OM to detect the protein level changes of the MAPK signalling pathway. The WB result showed the obvious elevation of the phosphorylation of ERK, JNK, P38 in VSMCs (dominantly by p‐ERK, P‐JNK) (Figure 5A). Besides, TGFβ is a known modulator of VSMCs phenotype, which critically affects SMC proliferation, differentiation, migration and vascular repair.^26^ The TGFβ signal is obviously downregulated in the human vessel wall of the superficial thrombotic vein.

The results of in vitro experiments also showed that the phenotypic switch of VSMCs occurred when SB505124 was applied to inhibiting the TGFβ signalling pathway. Previous studies have shown that TGFβ facilitates VSMCs differentiation by influencing the expression of SMC‐specific genes, which is required phosphorylation of Smad2 or Smad3.^27^ Consistent with previous reports, the increased expression of pSMAD2/3 was observed in VSMCs added with TGFβ2 under OM condition (Figure 5A). In the in vitro model, we firstly applied TNFα to induce VSMCs dedifferentiation and subsequently suppressed the TGFβ signalling pathway by SB505124 to accomplish the transdifferentiation of VSMCs to osteoblast‐like cells. We hypothesized that there is any crosstalk between the two signalling pathways, which is ultimately responsible for the appearance of the osteogenic phenotype of VSMCs. In addition to activating the canonical SMAD2/3 signalling pathway, TGFβ can also induce the activation of the non‐classical signalling pathway as MAPK signalling pathway.^28^ We employed the time‐course study to test the changes in the MAPK signalling pathway molecules by culturing VSMCs with TGFβ2 in OM. WB results demonstrated that TGFβ2 was also able to significantly raise the phosphorylation level of ERK. Meanwhile, incubation of TGF‐β2 only revealed a modest effect on phosphorylation level of JNK and p38 in rat VSMCs. Furthermore, some studies reported that TNFα could effectively induce the phosphorylation of SMAD2/3 in cells^29^, ^30^; our data indicated that p‐SMAD2,3 did not increase in VSMCs treated with TNFα in OM. The above results showed that the crosstalk between TNFα and TGFβ signalling pathways may be implemented through the activation of ERK. In addition, some other papers reported that JNK might involve in regulating the TGFβ signalling pathways.^31^ Thus, the phosphorylation levels of ERK and JNK in each group of the in vitro model were measured. WB results indicated that the expression of pERK1/2 and pJNK1 escalated in the SB group (Figure 5B), which implied that the activation of ERK and JNK MAPK pathway was possibly relevant to the regulation of VSMCs to the osteoblast‐like phenotype under the combined effect of TGFβ2 and TNFα.

### 3.6 Ameliorating effects of ERK signalling inhibition on TNFα activation and TGFβ blockage induced osteogenic phenotype of VSMC

To further verify our assumption, we separately added U0126 (inhibition of ERK/MAPK signalling pathway) and JNK inhibitor II (inhibition of JNK/MAPK signalling pathway) in the SB group to detect the changes in VSMC phenotype‐related genes. ERK (U0126) and JNK (JNK inhibitor II) inhibitors upregulated contractile phenotypic genes (*αSMA* and *Tagln*), but did not affect the synthetic phenotypic gene (*Col1a1*) (Figure 5C). Moreover, suppressing ERK in the SB group could slightly alleviate the increase of osteogenic phenotypic genes (*Runx2*, *Osx* and *Ocn*), but inhibiting JNK would aggravate the situation, where the osteogenic phenotypic genes (*Runx2*, *Osx* and *Ocn*) expression were significantly elevated (Figure 5C). Thus, we concluded that the combined effect of TGFβ and TNFα signalling pathways, regulating the osteoblast‐like phenotype switch of VSMCs, might be achieved by activating the ERK/MAPK signalling pathway.

We then established rat deep vein thrombosis (DVT) model^19^, ^32^ to mimic in vivo pathological conditions and delineate whether impaired TGFβ as well as increased TNFα signalling pathways could induce the osteogenic phenotype markers expression. F‐127 pluronic gel system was used to deliver compounds and/or recombinant proteins to the adventitial surface as previously described^33^ when venous thrombus was formed between day 14 and day 16 after inferior vena cava (IVC) ligation in DVT rats. As showed in Figure 5E, compared with control group, perivascular delivery of TNFα significantly increased the osteogenic phenotypic markers (*Runx2*, *Osx* and *Ocn*) expression. Meanwhile, we also observed osteogenic phenotypic markers, such as *Runx2*, increased by SB505124 treatment. Furthermore, when both TNFα and SB505124 were used to treat DVT, obviously enhanced the expression of osteogenic phenotypic markers and exhibited better homogeneity. This suggests impaired TGFβ as well as increased TNFα signalling pathways play predominant roles in inducing the osteogenic phenotype markers expression in DVT rats.

### 3.7 TGFβ2 compensation reduced TNFα activation and TGFβ blockage induced phenotypic switching and VSMCs calcification in vitro

In vitro model, SB505124 after TNFα stimulation in OM can induce dedifferentiation from the contractile phenotype to osteogenic phenotype in VSMCs. We assumed that the exogenous addition of TGFβ2 to activate TGFβ signalling might reduce the occurrence of the osteogenic phenotype of VSMCs. Thus, VSMCs cultured in OM were exposed to TNFα for 1 day, after which the medium was replaced with TGFβ2 rather than SB505124 to observe the expression of VSMCs phenotypic markers at the mRNA level. The qPCR analysis showed that compared with the SB group, the contractile (*αSMA* and *Tagln*) and synthetic (*Col1a1*) markers in the TGFβ2 group were distinctly increased, while the osteogenic phenotypic markers (*Runx2*, *Osx* and *Ocn*) showed an opposite pattern (Figure 5D). We concluded that after the stimulation with inflammatory factors, activating the TGFβ signalling pathway effectively alleviated the transformation of VSMCs to osteoblast‐like phenotype caused by the changes of TNFα and TGFβ signalling pathways in the early stage of PTS.

## 4 DISCUSSION

Vascular calcification (VC) is one of the most prevalent problems of ectopic calcification.^34^ VSMCs, the main cell type located in the medial layer of the vessel wall, have a critical role in regulating VC progress. After exposure to multiple growth factors and inflammatory mediators, VSMCs can adapt to several phenotypes, including adipogenic, macrophagic and calcific (osteogenic, chondrocytic and osteoclastic) phenotypes. During the dedifferentiation to osteogenic phenotype, VSMCs gradually lose contractile markers (while the osteogenic markers increase) and the capability of contraction and relaxation, which results in vasculature stiffness.^35^ In osteoblast‐like cells, the raised production of bone matrix proteins potentiates local calcification deposition and aggravates the symptom of vascular stiffness, which interferes with elasticity and compliance of the vessel wall.^36^ In this study, we further evaluated the calcium deposition within the venous wall, the cell type involved in the calcified remodelling of the venous wall after thrombosis and explored possible mechanisms in vitro.

We observed that obvious calcification deposition, located at the medial layer of a venous wall from thrombotic vein specimens, was co‐localized with αSMA and TAGLN (Figure 2C). This result implied that VSMCs participated in the calcified remodelling process of the venous wall after thrombosis and suggested that the osteogenic phenotype of VSMCs and calcium deposit within the medial venous wall may be an explanation of the abnormal changes in contraction and relaxation of the post‐thrombotic vein wall, which leads to venous stasis, venous hypertension, oedema and skin symptoms in the lower extremity.

Inflammation is the most obvious manifestation in the early phase of the thrombus. It promotes vascular recanalization and is responsible for vein wall injury. TNFα, the ‘master’ proinflammation cytokine, can induce VSMCs differentiation into synthetic phenotype, increasing proliferation and remodelling the extracellular matrix of the vessel wall.^37^ In addition, TNFα can also regulate the dedifferentiation of VSMCs into an osteoblast‐like phenotype to facilitate vascular calcification.^38^ Consistent with previous reports, our preliminary research found that the inflammatory factor TNFα was highly expressed within the superficial thrombotic vein. In order to clarify whether venous calcification after thrombosis was caused by TNFα, we initially treated VSMCs with TNFα alone in OM medium in vitro. qPCR results showed a downregulation of contractile phenotypic markers, but not increase in osteogenic phenotypic markers. Preliminary experiments suggested that although the inflammatory factor TNFα could effectively induce the dedifferentiation of VSMCs, it was not enough to induce the transformation of VSMCs into an osteoblast‐like phenotype.

There may be other factors involved in this process. TGFβ is a member of a highly evolutionary conserved secreted family, which consists of three TGFβ isoforms (TGFβ1, TGFβ2 and TGFβ3).^39^ TGFβ affects vascular development and disease via potentiating VSMCs differentiation and proliferation,^40^ while the effect of TGFβ on calcification is still controversial. When inhibiting TGFβ signalling in VSMCs by ablation TGFβR2, VSMCs reprogram into MSC‐like cells, and in turn, give rise to adipocytes, chondrocytes and osteoblasts, as well as into macrophage‐like cells.^41^ Some other studies reported that TGFβ contributes to vascular calcification by regulating VSMCs dedifferentiation into osteo/chondrogenic phenotype.^42^, ^43^ In our previous study, high expression of inflammatory factor TNFα was accompanied by significant downregulation of TGFβ2 within the venous wall after thrombosis. Based on this, after adding TNFα to induce dedifferentiation of VSMCs, SB505124 was equipped to block TGFβ signalling pathway, trying to induce the transformation of VSMCs into an osteoblast‐like phenotype. Our results revealed that the chronological application of TNFα and SB505124 promote the conversion of VSMCs to an osteoblast‐like phenotype and increase calcium deposition in vitro (Figures 3 and 4). When VSMCs replenished TGFβ2 after pretreated with TNFα for 1 day in OM medium, the contractile phenotypic markers were restored, combined with the inhibition expression of osteogenic markers. The above results proved that the blockage of the TGFβ signalling pathway after inflammatory response was one of the reasons for the occurrence of VSMCs osteogenic phenotype in the venous wall after thrombosis. It should be pointed out that the strategy involved in this in vitro calcification model well simulated the change of TNFα and TGFβ signalling pathways within venous calcification after thrombosis, which provided a good in vitro cell model to investigate the related molecular mechanisms of VSMCs calcification under such local cue.

Wnt/β‐catenin, BMP/SMAD1/5/8, RANK/RANKL and MAPK signalling influence the phenotype of VSMCs to regulate VC.^44^, ^45^, ^46^ Our data suggested that the inhibition of the TGFβ signalling pathway after inflammation to induce the transition of VSMCs into osteoblast‐like phenotype might be achieved by the activation of the ERK/MAPK signalling pathway (Figure 5B). Using U0126 to inhibit this signalling pathway could partially reverse the phenotype switch of VSMCs, suggesting a potential therapeutic target to prevent VSMCs dedifferentiation into osteogenic phenotype after thrombosis (Figure 5C). Although JNK/MAPK signalling pathway was also significantly activated in our results, there was no significant downregulation of osteogenic markers after the application of JNK inhibitor II. Yet, further research is required to clarify its relationship with the osteogenic phenotype of VSMCs.

**FIGURE 6:**
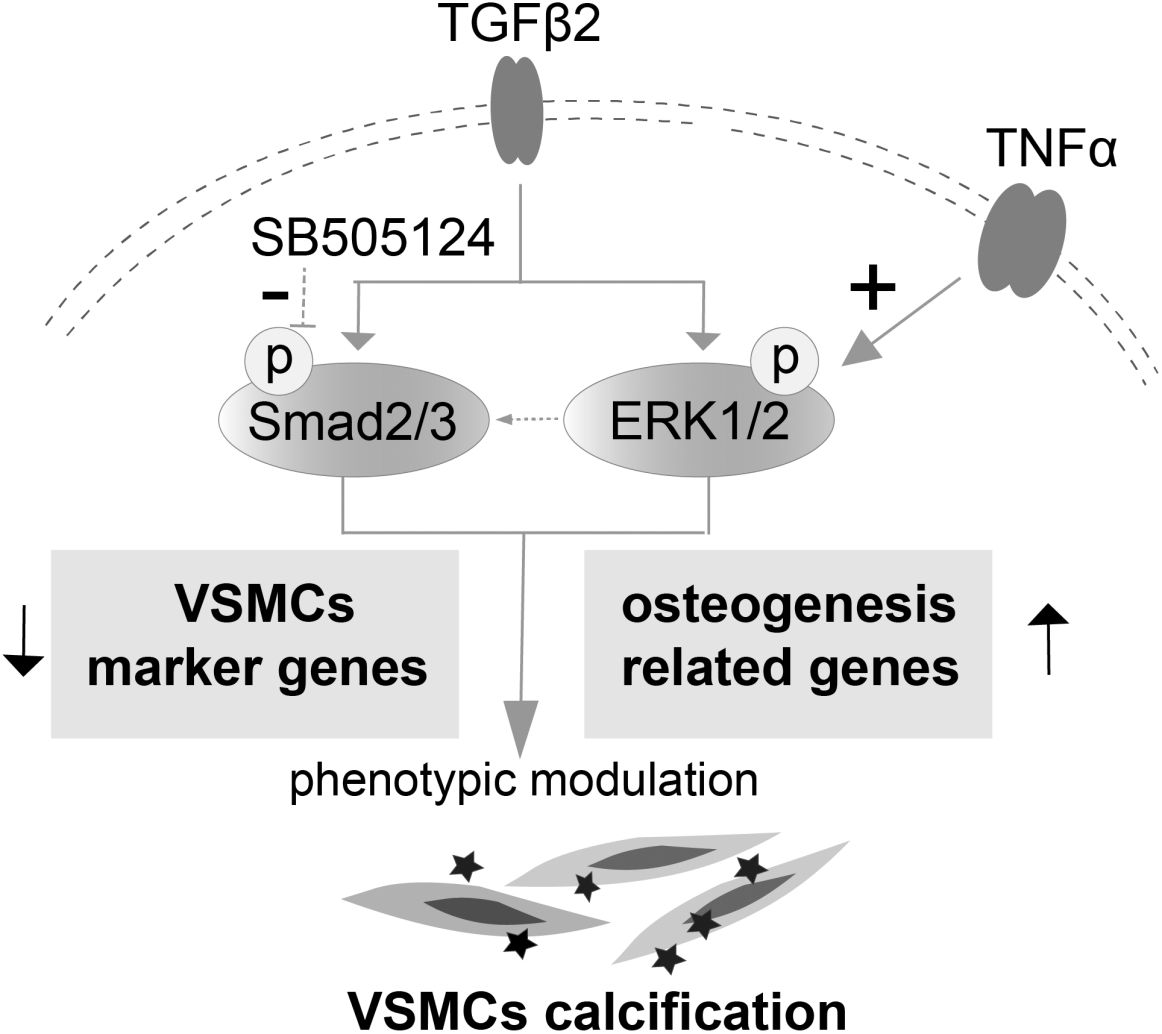
Schematic representation of TNFα activation and TGFβ blockage act synergistically to induce VSMCs calcification via non‐canonical TGFβ/ERK pathway

To sum up, our data indicated that venous calcification after thrombosis was closely related to the appearance of VSMCs osteoblast‐like cell phenotype. Moreover, our results showed that the inhibition of TGFβ signalling after stimulation by the inflammatory factor TNFα could promote the dedifferentiation of VSMCs into osteoblast‐like cells and the activation of the ERK/MAPK signalling pathway was responsible for this process (Figure 6). In addition, after TNFα stimulation, supplementing TGFβ2 or inhibiting ERK/MAPK signalling pathway partially inhibited the appearance of VSMCs osteogenic phenotype. Our findings may provide new insight on the early prevention of venous calcification after thrombosis. In fact, JNK/MAPK signalling pathway seems to regulate VSMCs calcification in our working model, while its working pattern is different from ERK/MAPK signalling. However, this remains to be further investigated.

## AUTHOR CONTRIBUTIONS

**Penghui Wang:** Formal analysis (equal); investigation (equal); validation (equal); visualization (equal); writing – original draft (equal). **Yiqing Pan:** Data curation (equal); formal analysis (equal); methodology (equal); visualization (equal); writing – original draft (equal). **Chenghao Yang:** Investigation (equal); methodology (equal); validation (equal). **Linjie Zhang:** Investigation (equal); validation (equal); visualization (equal). **Zhen Zhao:** Data curation (equal); investigation (equal); methodology (equal). **Kaichuang Ye:** Funding acquisition (supporting); investigation (equal); methodology (equal); validation (equal). **Lei Li:** Investigation (equal); methodology (equal); validation (equal). **Shoubing Xia:** Investigation (equal); validation (equal); visualization (equal). **Xinwu Lu:** Project administration (equal); supervision (equal). **Huihua Shi:** Investigation (equal); methodology (equal); validation (equal). **Weimin Li:** Methodology (equal); project administration (supporting); supervision (equal). **Minyi Yin:** Conceptualization (lead); funding acquisition (equal); project administration (lead); supervision (equal); writing – review and editing (lead).

## CONFLICT OF INTEREST

No conflicts of interest, financial or otherwise are declared by the authors.

## DATA AVILABILITY STATEMENT

All relevant data from this research are available from the corresponding author upon a reasonable request.

## Supporting information


Table S1

Table S2

Table S3
