# Supplementary material for: TNFα activation and TGFβ blockage act synergistically for smooth muscle cell calcification in patients with venous thrombosis via TGFβ/ERK pathway
Source: J Cell Mol Med. 2022 Jul 8;26(16):4479–91. doi: 10.1111/jcmm.17472 (PMC9357635; doi:10.1111/jcmm.17472)
Supplement: Supplementary file 1 — Table S1 [file JCMM-26-4479-s002.docx]

**Table S1. Demographics of patients with venous** **thrombosis.**

| Variable | | Patients |
| --- | --- | --- |
| Age (years), y±SEM | | 63.4 ± 3.7 |
| Gender (Men/Women) | | 4/7 |
| Race/ethnicity, n | | Asian (11) |
| Blood Leukocyte count (×10^9^/L), y±SEM | | 5.00 ±0.35 |
| Neutrophil percentage (%±SEM) | | 59.28± 1.85 |
| Medical history,  n (%) | Hypertension | 5 / 11 (45.4) |
|  | Diabetes mellitus | 0 / 11 (0) |
|  | High cholesterol | 3 / 11 (27.2) |
|  | Prior thrombosis history | 0 / 11 (0) |
|  | Smoker | 0 / 11 (0) |
| Active medical conditions in the last month,  n (%) | Infectious / inflammatory | 0 / 11 (0) |
|  | Stroke | 0 / 11 (0) |
