# Supplementary material for: TNFα activation and TGFβ blockage act synergistically for smooth muscle cell calcification in patients with venous thrombosis via TGFβ/ERK pathway
Source: J Cell Mol Med. 2022 Jul 8;26(16):4479–91. doi: 10.1111/jcmm.17472 (PMC9357635; doi:10.1111/jcmm.17472)
Supplement: Supplementary file 2 — Table S2 [file JCMM-26-4479-s003.docx]

**Table S2. Detailed information of antibodies.**

| Name | Company | Number |
| --- | --- | --- |
| αSMA | Abcam | Ab7817 |
| TAGLN | Abcam | Ab10135 |
| COL1A1 | Cell Signaling Technology | #91144 |
| RUNX2 | Cell Signaling Technology | #12556 |
| Phospho-ERK1/2 (Thr202/Tyr204) | Cell Signaling Technology | #4377 |
| Total-ERK2 | Santa Cruz Biotechnology | sc-154 |
| phospho-JNK (Thr183/Tyr185) | Cell Signaling Technology | #4668 |
| phospho-P38 (Thr180/Tyr182) | Cell Signaling Technology | #9216 |
| total-P38 | Cell Signaling Technology | #9212 |
| phospho-Smad2 (Ser465/467) /Smad3 (Ser423/425) | Cell Signaling Technology | #8828 |
| SMAD2/3 | Cell Signaling Technology | #8685 |
| α-tubulin-HRP | Proteintech | HRP-66031 |
