# Supplementary material for: TNFα activation and TGFβ blockage act synergistically for smooth muscle cell calcification in patients with venous thrombosis via TGFβ/ERK pathway
Source: J Cell Mol Med. 2022 Jul 8;26(16):4479–91. doi: 10.1111/jcmm.17472 (PMC9357635; doi:10.1111/jcmm.17472)
Supplement: Supplementary file 3 — Table S3 [file JCMM-26-4479-s001.docx]

Table S3a. Sequence of human primers used in qPCR

| Gene symbol | Forward primer sequence (5’-3’) | Reverse primer sequence (5’-3’) |
| --- | --- | --- |
| *RUNX2* | GCCTTCCACTCTCAGTAAGAAGA | GCCTGGGGTCTGAAAAAGGG |
| *OSX (SP7)* | CCTCTGCGGGACTCAACAAC | AGCCCATTAGTGCTTGTAAAGG |
| *ALPL* | GGGACTGGTACTCAGACAACG | GTAGGCGATGTCCTTACAGCC |
| *OCN (BGLAP)* | CACTCCTCGCCCTATTGGC | CCCTCCTGCTTGGACACAAAG |
| *GAPDH* | TGCACCACCAACTGCTTAGC | GGCATGGACTGTGGTCATGAG |

Table S3b. Sequence of rat primers used in qPCR

| Gene symbol | Forward primer sequence (5’-3’) | Reverse primer sequence (5’-3’) |
| --- | --- | --- |
| *αSMA (Acta2)* | CGCCATCAGGAACCTCGAGA | CAAAGCCCGCCTTACAGA |
| *Tagln (SM22α)* | CCACAAACGACCAAGCCTTTT | CGGCTCATGCCATAGGATG |
| *Col1a1* | ATCTCCTGGTGCTGATGGAC | ACCTTGTTTGCCAGGTTCAC |
| *Runx2* | CACAAGTGCGGTGCAAACTT | AATGACTCGGTTGGTCTCGG |
| *Osx (Sp7)* | GCTCACTATGGCTCCAGTCC | GGGGCTGAAAGGTCAGTGTA |
| *Ocn (Bglap)* | GCCCTGACTGCATTCTGCCTCT | TCACCACCTTACTGCCCTCCTG |
| *Gapdh* | GACAACTTTGGCATCGTGGA | ATGCAGGGATGATGTTCTGG |
